# Family Spirit Nurture (FSN) – a randomized controlled trial to prevent early childhood obesity in American Indian populations: trial rationale and study protocol

**DOI:** 10.1186/s40608-019-0233-9

**Published:** 2019-05-06

**Authors:** Allison Ingalls, Summer Rosenstock, Reese Foy Cuddy, Nicole Neault, Samantha Yessilth, Novalene Goklish, Leonela Nelson, Raymond Reid, Allison Barlow

**Affiliations:** Center for American Indian Health, Department of International Health, Johns Hopkins Bloomberg School of Public Health, Baltimore, MD USA

**Keywords:** Childhood obesity, American Indian, Pregnancy and childbirth, Parenting, Randomized controlled trials, Home-visiting, Prevention

## Abstract

**Background:**

Childhood overweight and obesity is a persistent public health issue in the US. Risk for obesity and obesity-related morbidity throughout the life course begins in utero. Native Americans suffer the greatest disparities in the US in childhood overweight and obesity status of any racial or ethnic group. Existing early childhood home-visiting interventions provide an opportunity for addressing obesity during the first 1000 days. However, to date, no evidence-based model has been specifically designed to comprehensively target early childhood obesity prevention.

**Methods:**

This study is a randomized controlled trial to test the efficacy of home-visiting intervention, called Family Spirit Nurture, on reducing early childhood obesity in Native American children. Participants are expectant Native American mothers ages 14–24 and their child, enrolled from pregnancy to 24 months postpartum and randomized 1:1 to receive the Family Spirit Nurture intervention or a control condition. The intervention includes 36 lessons delivered one-on-one by locally-hired Native American Family Health Coaches to participating mothers from pregnancy until 18 months postpartum. A mixed methods assessment includes maternal self-reports, maternal and child observations, and physical and biological data collected at 11 time points from 32 weeks gestation to 2 years postpartum to measure the intervention’s primary impact on maternal feeding behaviors; children’s healthy diet and physical activity; children’s weight status. Secondary measures include maternal psychosocial factors; household food and water security; infant sleep and temperament; and maternal and child metabolic status.

**Discussion:**

None of the 20 current federally-endorsed home-visiting models have demonstrated impacts on preventing early childhood obesity. The original Family Spirit program, upon which Family Spirit Nurture is based, demonstrated effect on maternal and child behavioral health, not including obesity related risk factors. This trial has potential to inform the effectiveness of home-visiting intervention to reduce obesity risk for tribal communities and other vulnerable populations and expand public health solutions for the world’s obesity crisis.

**Trial registration:**

Clinicaltrials.gov (Identifier: NCT03334266 - Preventing Early Childhood Obesity, Part 2: Family Spirit Nurture, Prenatal - 18 Months; Retrospectively registered on 07 November 2017).

## Background

Urgent recent studies have countered recent reports suggesting overall prevalence of childhood overweight and obesity has plateaued in the US [1, 2] and have pointed to an alarming rise in moderate to severe obesity in children 2–5 years of age [1]. Increased risk for obesity begins early, starting in utero. Suboptimal in-utero risks have been associated with early metabolic dysregulation, altered body composition (i.e. less lean muscle and more adipose tissue), and fetal structural changes which increased risk for development of Type 2 diabetes, cardiovascular disease (CVD), non-alcoholic fatty liver disease and cancer [3–7]. A greater percentage of Native American (NA) women give birth to large for gestational age babies than women of all other races/ethnicities [8]. Obesity disparities among NA children persist from birth through early childhood, adolescence, and adulthood. Nearly one-third of all NA youth 2 to 19 years old are obese, with a prevalence of 20.7% for children 2 to 5 years of age [9]. While the prevalence of overweight NA youth has decreased over time, the prevalence of obesity has risen, indicating an increase in the severity of the problem [9]. NA adults have the highest age-adjusted rates of obesity (43.7%) and diabetes (17.6%) among all racial groups in the US [10].

Early life obesity translates to formidable chronic disease—especially CVD and diabetes [11]. CVD, once rare among NAs, now exceeds rates in other US populations and is more often fatal [12]. Further, diabetes has emerged as a public health issue among NA youth. In 2009, diabetes rates among NA youth 10–19 years of age were 2.6 times higher than the US all races/ethnicities rate and 7.0 times higher than the non-Hispanic White youth rate [13]. The consequences of childhood overweight and obesity extend beyond children’s health risks to poorer academic achievement, psychosocial functioning, and economic loss [14–16]. As the challenges of childhood obesity disproportionately affect lower-income and racial and ethnic minority populations, prevention research with NA populations can yield innovations with applicability for other high-risk, low-resource communities.

### Role of infant and young child feeding practices in childhood obesity

National surveys indicate US infants: 1) consume excess calories— particularly from sugar sweetened beverages (SSBs), snacks and desserts; 2) are breastfed briefly, non-exclusively, or not at all; and 3) receive complementary foods and/or beverages before six months of age, all of which are risk factors for obesity later in life [17–25].

### Sugar sweetened beverages

SSBs are the largest source of added sugar in US children’s diets and play a key role in early childhood obesity, particularly among NA children [26, 27]. Data support that children introduced to SSBs before 6 months of age are 92% more likely to be obese at age 6. At age 10–12 months, those who consume > 3 SSBs per week have twice the obesity rate compared to those who consume no SSBs [21]. Overweight/obese 2- to 5-year-old NA children consume 51% more SSBs than their normal-weight NA counterparts [28]. SSB consumption has also been linked to other negative health outcomes including dental caries, insulin resistance, headaches, and other caffeine-related ailments [29]. In a prior home-visiting trial conducted in Southwestern tribal communities, 46% of NA mothers fed SSBs to infants by 6 months of age, and 87% by 12 months. In a recent systematic review examining strategies to reduce sugary drink consumption among 0- to 5-year-olds, authors called for more research in this area with understudied groups, including NAs. They also urged for further research in developing an evidence base for reducing SSB consumption among children 0–5 in non-preschool/daycare settings [30].

### Breastfeeding

Breastfeeding protects against obesity, CVD, and diabetes [31, 32]. National data indicate that less than three-quarters (68.9%) of NA mothers initiate breastfeeding and only a little more than one-third (37.1%) continue through 6 months postpartum [33]. NA mothers have reported lack of understanding of obesity-related benefits of breastfeeding [34]. Additionally, there is an identified need for more research about how breastfeeding education and support may impact uptake of breastfeeding and duration [35].

### Introduction of complementary foods

Studies indicate early introduction of complementary foods (< 6 months of age) increases infants’ risk of obesity [31]. In addition, studies of NA mothers have indicated lack of understanding of the proper timing of introduction to complementary foods [34]. It is also well-known that early dietary patterns (before 24 months postpartum) impact lifelong food preferences [36]. The types of food offered and the feeding environment shape children’s lifelong eating habits [36].

### Responsive feeding practices

In addition to providing children with healthy foods, parenting and feeding practices are key to obesity prevention [36]. Responsive parents recognize infant/young child communication signals and respond in a sensitive (prompt, emotionally-supportive [non-intrusive], developmentally-appropriate) manner. In the context of feeding, responsivity refers to parental recognition of child signals of hunger and satiety, coupled with a sensitive response [37, 38]. Non-responsive feeding (e.g. forceful or indulgent feeding) is associated with early rapid weight gain in the first 6–12 months of life and predicts early childhood obesity [38–42]. Expected benefits of responsive feeding include children’s increasing attention to internal signals of hunger and satiety [14–16, 42–46] and reduced risk of rapid weight gain and pediatric obesity [38].

### Role of physical activity in childhood obesity

Early childhood is an important time for establishing positive health behaviors to prevent obesity, including a physically-active lifestyle. Physical activity (PA) guidelines for US toddlers recommend at least 30 min/day of structured PA and 60 min/day of unstructured PA, for a total of 90 min/day [47]. A recent study found that low-income, racially-diverse toddlers are not meeting this recommendation [48].

### Implementation of Family Spirit Nurture

It is widely recognized that intervention during the first 1000 days is an important strategy for reducing risk for obesity and obesity-related morbidities over the life course [49–51]. A recent systematic review identified several risk factors during the first 1000 days that are associated with later childhood overweight, including excess maternal weight gain during pregnancy, higher maternal pre-pregnancy body mass index (BMI), gestational diabetes, low socioeconomic status (SES), high infant birth weight, and rapid infant weight gain in the first 6 and 12 months of life [39]. Another systematic review found that even the existing childhood obesity research studies beginning in pregnancy do not extend beyond the first months of life. Authors of this review urge researchers to examine outcomes until at least two years postpartum [52].

This study will assess the impact of a home-visiting program, called Family Spirit Nurture (FSN), on reducing early childhood obesity in NA children during this critical developmental time period, from pregnancy to age 2, with potential to impact obesity and obesity-related morbidities over the life course. The FSN intervention is delivered from pregnancy until 18 months postpartum (with follow-up through 24 months postpartum) and is built on a strong platform: the evidence-based Family Spirit (FS) home-visiting program model, designed and evaluated by the Johns Hopkins Center for American Indian Health (JHCAIH) with tribal communities over a 20-year period [53, 54]. Over 120 tribal communities and 2 non-Native communities across 20 states have been trained to deliver the FS model. The current study integrates new curriculum content and activities into FS that are directed at obesity prevention. In addition, this study examines how the home-visiting behavior change intervention impacts underlying metabolic health through collection of maternal and infant blood samples.

Primary research questions include: 1) Is the intervention effective in increasing mothers’ likelihood of meeting breastfeeding and complementary feeding recommendations?; 2) Does the intervention improve responsive parenting/feeding behaviors?; 3) Is the intervention effective in decreasing children’s consumption of SSBs, snacks and desserts, and increasing consumption of age-appropriate fruits and vegetables?; 4) Is the intervention effective in increasing children’s physical activity levels and decreasing children’s screen time and other sedentary activities?; and 5) Does the intervention improve children’s BMI z-scores? Secondary research questions include: 1) Do maternal psychosocial factors and household food/water security and/or constrained PA environments moderate intervention impacts on: infant and young children’s feeding behaviors and infant and young children’s diets, PA patterns, and weight status?; 2) How do maternal/infant characteristics, diet, and behaviors impact the underlying biologic mechanisms of early childhood obesity?; and 3) Can social and behavioral interventions impact infant metabolic health?

The aim of this paper is to describe the Family Spirit Nurture intervention and the randomized controlled trial evaluating its efficacy.

## Methods/design

### Trial design

**Fig. 1:**
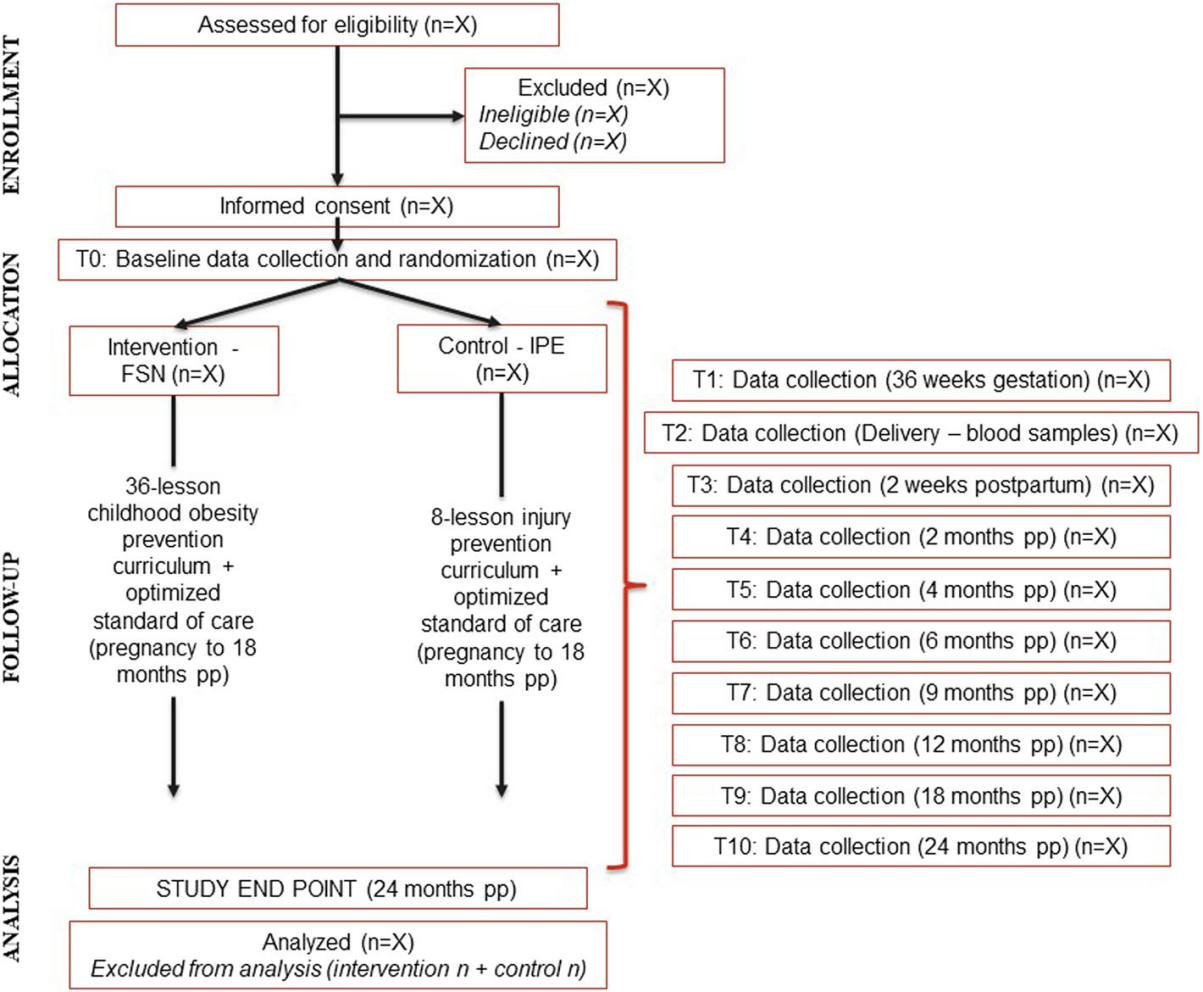
Study Design and Data Collection

This study is a two-arm randomized controlled trial (RCT) with primary endpoints of parent feeding practices, young children’s diet and PA, and early childhood (0–2 years of age) weight status. Stratified block randomization with a 1:1 allocation is used to ensure equal allocation to intervention and control groups across key maternal baseline characteristics (Fig. 1).

### Participants

The target population in this study is expectant NA mothers ages 14–24 who are having their first or second baby. Compared to the general US population, a high rate of NA women begin childbearing in their teenage years [55]. From 2016 to 2017, 25.8% of NA mothers were under the age of 20 when their first child was born compared to 11.5% of mothers for the US all races population (2.24 times higher). In addition, 5.8% of NA mothers were under the age of 20 when their second child was born compared to 2.3% of mothers for the US all races population (2.52 times higher) [55]. The participating communities have long seen the need for family strengthening interventions for young parents, and prior Family Spirit trials that are the foundation for this study have targeted young parents: two trials included mothers less than 20 years old, and another included mothers less than 22 years old [54, 56, 57]. By focusing on young parents with low parity, the intervention can achieve the greatest public health impact as what parents learn early in their reproductive life may be applied to future pregnancies and child-rearing. The majority of early childhood home-visiting interventions throughout the world--and those with most evidence base in US [58]--focus on young, low-parity mothers to optimize use of resources to promote family-based health outcomes.

Three reservation-based NA communities in the US are participating in this trial: two large Navajo communities and the Fort Apache Indian Reservation.

### Navajo Nation

The Navajo Nation covers the corners of three states: Arizona, New Mexico, and Utah and is about the size of West Virginia. The Navajo Nation is the largest reservation in the United States, covering 27,673 square miles. There are approximately 175,000 people living on the Navajo Nation and 33% of all tribal members are under the age of 18. The average household size on the Navajo Nation is 3.5 persons. The median household income for the Navajo Nation is $27,389 and 14.7% of the households are multi-generational. Poverty rates on the Navajo Nation (38%) are more than twice the poverty rate in Arizona (15%). Almost half (44%) of children < 18 years are living in poverty. In a recent study on Navajo Nation examining water insecurity, 37.2% of households reported having inadequate access to safe drinking water. With just 13 grocery stores across the entire Navajo Nation, many community members are also food insecure.

### Fort Apache Indian Reservation

The Fort Apache Indian Reservation is home to ~ 17,500 White Mountain Apache Tribe (WMAT) members, with nearly half < 20 years of age [59]. The reservation encompasses 1.7 million acres along eastern Arizona. The WMAT endures notable demographic and environmental challenges that impact behavioral risks among youth, including a 40% unemployment rate [60]. The median household income is $26,973, half that of the State ($51,310). More than half of children under 18 are considered as living in poverty. About 40% of WMAT households are led by single mothers [59].

### Recruitment

The study is recruiting *N* = 338 expectant NA mothers who: 1) are between the ages of 14 and 24 at conception, 2) are less than 32 weeks gestation at consent, 3) have one or no other children, and 4) live within 50 miles of the Indian Health Service (IHS) medical facility in each participating community. Participants must be willing to undergo random assignment and participate in all aspects of the study. All participants must complete informed consent to participate. Participants under age 18 must have parent/guardian consent. The first participant was consented on October 16, 2017, and the first randomization occurred on December 1, 2017.

We are using non-probability sampling to recruit participants. Through an approved waiver of HIPAA Authorization, study staff obtain demographic information from potential participants’ electronic health records and clinic visit appointment logs at participating health clinics. This allows them to directly contact potentially eligible participants. Participants are recruited through local health and social service agencies (i.e., OB/GYN clinics, WIC programs, etc.), through public postings in community gathering spots (i.e., grocery stores, clinics, WIC offices, schools, etc.), through public service announcements on the local radio, and by print advertising in the local newspaper. Participants are also recruited during public gatherings (i.e., health fairs, job fairs, etc.). We are recruiting participants over a 24-month period (Fall 2017-Fall 2019).

### Randomization

Participants are individually-randomized to receive the intervention or control program. Randomization is completed after the completion of the baseline assessment. Randomization is stratified by age (< 18 years old and 18 years and older), site, and food and water security status. Randomization lists for each site were created and uploaded to REDCap prior to study initiation using STATA 14 statistical software [61]. The randomization lists are not accessible by study staff. After baseline is complete, the field coordinator verifies baseline data and randomizes the participant in REDCap. Study staff are then informed of the participant’s study allocation. This is locked and unchangeable within REDCap.

### Ethical considerations

This study was reviewed and approved by Health Boards and Chapter Houses in the participating Navajo communities, White Mountain Apache Tribal Council and Health Board, IHS Whiteriver Service Unit, Navajo Nation Institutional Review Board, the Phoenix Area IHS Institutional Review Board, and the Johns Hopkins Bloomberg School of Public Health Institutional Review Board (Current protocol version: #11, approved on September 12, 2018). Any protocol modifications are submitted as an amendment to relevant IRBs. Supervisors then inform study staff and conduct additional trainings as needed. If protocol modifications are significant, trial registries are updated.

Serious adverse events (e.g., participant death or hospitalization) for both mothers and/or children are reported on a real-time basis to participating IRBs, and tabulated and reported to the trial’s Data Safety and Monitoring Board (DSMB). The three-member DSMB meets with study investigators biannually to review study progress and adverse events. The data manager unblinds adverse events for the DSMB, but other study staff do not have access to the unblinded report. DSMB members include a senior behavioral health trials expert, a tribal (Navajo) health leader with tribal research ethics expertise, and a biostatistics expert. If the DSMB were to determine the intervention in part or whole were causing harm to participants, the intervention would be modified accordingly or discontinued.

### Intervention: Family Spirit Nurture (FSN)

**Fig. 2:**
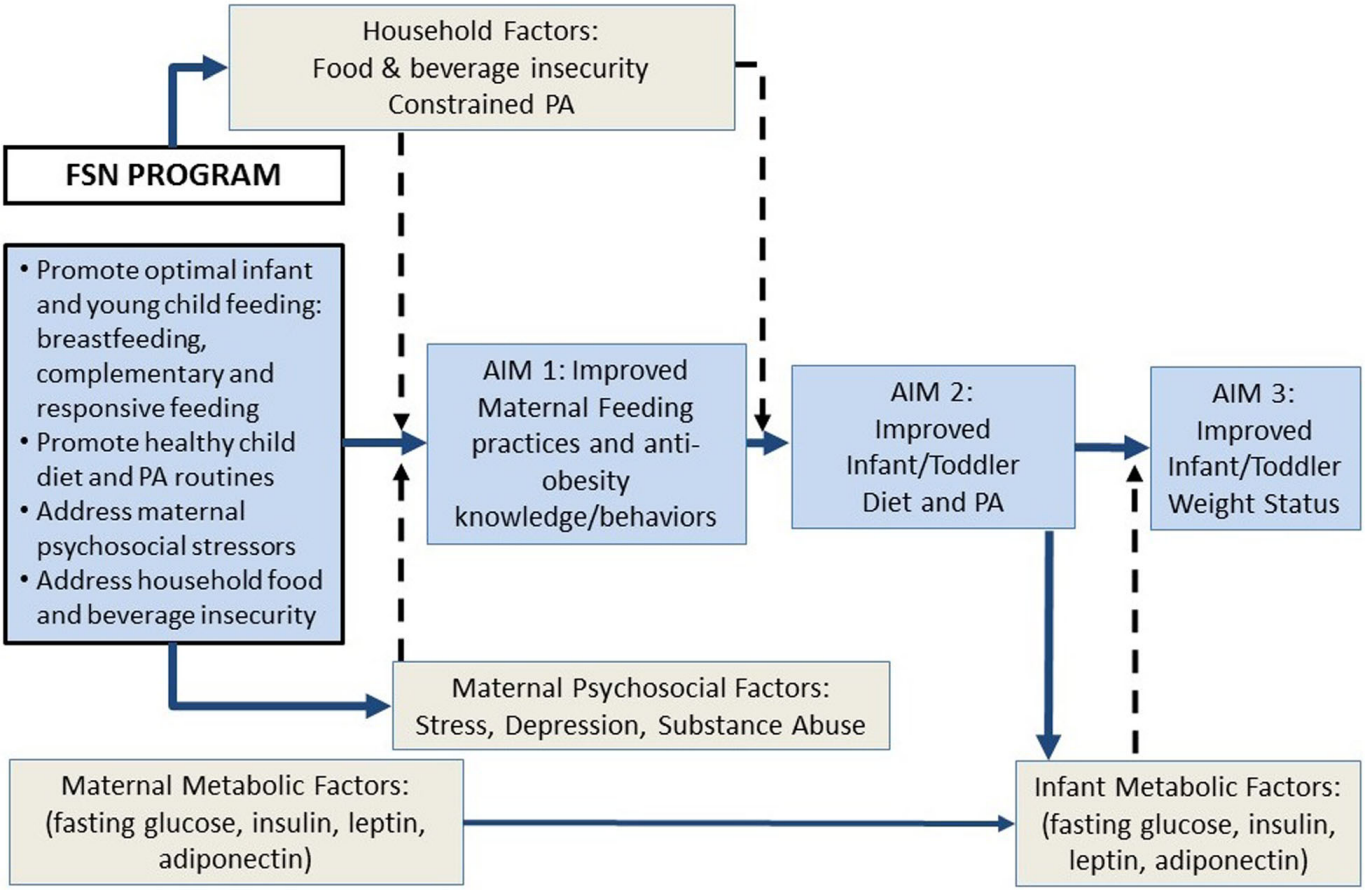
FSN Conceptual Model

The FSN conceptual model (Fig. 2) is based on G.R. Patterson’s family systems ecological developmental theory [62], which underpinned the original FS intervention design [63]. The conceptual model posits specific early childhood parenting behaviors (i.e., breastfeeding and optimal complementary and responsive feeding) as the central influence on infant/toddlers’ obesity-related outcomes [63]. The model also accounts for maternal psychosocial factors and key household-level factors that potentially moderate uptake of targeted parenting behaviors, if unaddressed. Therefore, FSN is designed to target modifiable components of the family system hypothesized to reduce early childhood obesity. Primary intervention aims are to promote mothers’ optimal breastfeeding, complementary and responsive feeding, child’s diet and physical activity practices, and children’s healthy weight status. Secondary aims are to: 1) address maternal psychosocial (i.e., stress, depression, substance use) and household environmental factors (food/water insecurity, constrained physical activity) that would otherwise impede mothers’ ability and motivation to implement knowledge and behaviors targeted by FSN education; and 2) to explore whether maternal and infant biologic factors (e.g., metabolic dysregulation) moderate intervention impact, and whether infant biologic factors are modifiable by the FSN intervention. In addition, based on social cognitive theory [64], the culturally-matched, empathetic Family Health Coaches (FHC) who are teaching the FSN curriculum and modeling positive normative behaviors are expected to further promote mothers’ motivation to adopt behaviors promoted by FSN.

#### Curriculum content and educational process

FSN curriculum development was initiated through an iterative process between Johns Hopkins University and communities in the Navajo Nation, as part of an earlier project. For the current study, this participatory approach was continued and included leadership from both Navajo and WMAT communities for cultural and contextual guidance and continuous engagement of stakeholders. A series of community and staff meetings with Native home visitors and stakeholders (one-on-one and group) guided key curriculum content and ensured inclusion of cultural and community-specific beliefs and practices around infant and young child feeding and physical activity. The 36 FSN lessons now comprise three key content domains: 1) promotion of responsive parenting and responsive feeding practices; 2) promotion of optimal infant and young child feeding practices and physical activity; and 3) promotion of maternal psychosocial well-being and safe and reliable home environment (Table 1).

**Table 1:** FSN Intervention Components

	Period (Frequency)	Responsive Parenting & Responsive Feeding	Infant and Young Child Feeding (IYCF) and PA	Maternal & Child Well-being and Home Environment
28 weeks gestation	Prenatal (biweekly)	• Introduction: fetal & child growth, development, & behaviors	• Prenatal nutrition & PA • Breastfeeding basics	• Planning for a healthy delivery
34 weeks gestation	Prenatal (biweekly)	• Introduction: responsive parenting/ feeding	• Prenatal nutrition & PA goal-setting • Breastfeeding promotion	• Baby care basics/ making a safe home for play
Birth	Neonatal (weekly)	• Normal growth, feeding, & sleeping patterns • Recognition of hunger/ satiety cues	• Feeding support visit • Importance of exclusive breastfeeding (or formula feeding if breastfeeding not possible)	• Family planning options • Daily routines for healthy living
1 month post-partum	Early infancy 1 (weekly)	• Understanding baby’s needs & behaviors • Managing fussy behavior • Responding quickly & appropriately to behavior cues • Discussing reduced screen time	• Promotion of exclusive breastfeeding (or formula feeding if breastfeeding not possible) • Feeding during illness • Child opportunities to explore through touch, movement, sight, & sound (tummy time)	• Resisting drug use • Conflict/ communication skills with baby’s father and/or family/ household members • Involving fathers and other caregivers in baby’s care
3 months post-partum	Early infancy 2 (biweekly)	• Play & communication activities • Looking/ smiling at child during feeding • Responding to baby’s cues and gestures • Establishing healthy sleep routines • Reducing exposure to screen time	• Promotion of exclusive breastfeeding (or formula feeding if breastfeeding not possible) • Gross & fine motor play • Continued tummy time • Monitoring patterns/ reducing screen time	• Skills for healthy living: handling stress, positive thinking, addressing home food, beverage, & PA environment
6 months post-partum	Mid-infancy (monthly)	• Play & communication activities (talking, singing, imitation) • Providing consistent routine • Eating with few distractions • Finger foods for child to touch	• Continued breastfeeding and/or formula feeding, along with complementary feeding (sequence) • Types/ textures of complementary foods • Increasing healthy diet variety • Gross & fine motor play	• Revisiting family planning/ sexual health (STI knowledge & testing) • Safe & reliable play spaces • Meal planning
9 months post-partum	Late infancy (monthly)	• Play & communication activities (“bye-bye”, imitation) • Singing to child during daily activities • Eating with child (modeling healthy eating) • Updating routines as needed	• Helping infant transition to healthy family lifestyle (diet & PA) • Ensuring infant is hungry at meals (avoid SSBs, snacks, desserts) • Continued breastfeeding and/or formula feeding	• Skills for healthy living: saying “no”, healthy relationships, problem-solving, positive thinking, accessing resources for a positive food & beverage environment
12 to 18 months post-partum	Toddlerhood (monthly)	• Monitoring toddler routines • Maintaining consistent routines (sleeping, eating) • Play & communication activities (reading, playing games) • Modeling healthy eating • Self-feeding • Avoiding food as a reward	• Giving toddler fixed “healthy” choices (e.g. banana or orange) • Transitioning to a cup • Dealing with food refusal and pickiness • Promoting PA/ reduced screen time • Involving toddler in food shopping & preparation • Providing daily opportunities for gross motor play	• Budgeting for family’s needs • Confident & effective parenting (consistent rules) • Toddler care practices/ making a safe home for play • Safe/ consistent alternative caregivers

The American Academy of Pediatrics’ (AAP) Caring for Your Baby and Young Child: Birth to Age 5 [65] was selected as the definitive reference for child care and parenting information and was compatible with tribal and IHS standards of care. The World Health Organization’s (WHO) “Guiding Principles for Complementary Feeding of the Breastfed Child” [66] was the primary reference for young child feeding content.

Family Health Coaches (FHCs) teach mothers the FSN curriculum in a one-on-one format using tabletop flipcharts in participants’ homes or other private locations. Lessons are timed to match children’s development and are highly visual and interactive, with drawings by Native artists and stories/scenarios to illustrate key points. FHCs are trained to use motivational interviewing techniques and the “VISION” tool (Visualize goal; Identify sub-goals, Set timeline, Identify barriers, Overcome roadblocks, Nurture sources of support) pretested by previous Family Spirit trials to help participants set short- and long-term goals and troubleshoot barriers to goals. As a brief example, within the Infant and Young Child Feeding domain**,** FHCs educate and illustrate optimal feeding practices and conduct activities teaching mothers how to feed their baby and read and respond to hunger and satiety cues. They use the VISION tool at the end of each lesson to help mothers set goals related to feeding practices and monitor progress on these goals at the beginning of each subsequent lesson.

#### Home-visit structure [63]

Each home visit is structured to include: a) warm-up period, b) review of the last lesson, c) check on past referrals, d) teaching lesson content, e) activities that model/practice lesson content and help set goals, f) participant review and summary of key lesson points, g) question and answer period, h) plans for homework or referrals, and i) setting up next appointment.

#### Lesson frequency and duration

FSN consists of 36 comprehensive lessons delivered bi-weekly from 28 weeks gestation until birth, weekly from birth to 3 months postpartum, bi-weekly from 3 to 6 months postpartum, and monthly from 6 to 18 months postpartum. Each visit is expected to take up to 60 min. Dosage is based on past evidence that effective home-visiting programs have generally planned for ~ 60 visits over a 1- to 5-year period and aimed to deliver between 32 and 56% of these visits (range 22–33 visits) [67]. Past Family Spirit trials established feasibility of proposed schedule [63].

### Control condition

#### Injury prevention education (IPE)

Mothers randomized to the control condition receive eight educational lessons on the following injury prevention topics: 1) vehicle safety for infants and mothers; 2) scald burns; 3) smoke alarms; 4) home safety; 5) poison storage; and 6) animal bites. These topics are supported by epidemiological data around prevalent injuries in the participating communities. NA populations, including study participants, experience injury disparities, particularly in early childhood. Thus, the control condition was selected as ecologically meaningful to community well-being, while non-contaminating to the intervention condition. Injury prevention education provided to the control group is kept completely separate from the intervention education. Lessons are delivered in the same format as the FSN lessons, by trained Family Health Liaisons (FHLs) in the home of the participant or in a private place of their choosing.

#### Lesson frequency and duration

The IPE lessons occur at the same time points as the following assessments: 36 weeks gestation, 2 weeks postpartum, and at 2, 4, 6, 9, 12 and 18 months postpartum. Each lesson visit is expected to take up to 30 min.

### Optimized standard of care (OSC)

Both the intervention and the control groups will receive OSC, which consists of transportation assistance to prenatal appointments (up to 6) and well-baby clinic appointments (up to 8) as recommended by the IHS and AAP. Both FHCs and FHLs provide rescue services through linkages to community agencies as needed. They often act as advocates for the mothers and families they serve to connect them to needed resources such as housing and food and nutrition assistance.

### Data collection time points

Our mixed-methods assessment includes maternal self-reports and interviews administered using Research Electronic Data Capture (REDCap) tools hosted at Johns Hopkins University, observations (including maternal/infant anthropometric measurements, infant accelerometry, and home safety assessments), maternal/infant medical chart reviews and maternal/infant blood sample collection (Table 2). REDCap is a secure, HIPAA compliant, web-based application designed to support data capture for research studies. It provides 1) an intuitive interface for validated entry; 2) audit trails for tracking data manipulation and export procedures; 3) automated export procedures for seamless data downloads to common statistical packages; and 4) procedures for importing data from external sources [68].

**Table 2:** List of Evaluation Measures

		Pregnancy		Postpartum							
		Baseline	36 weeks gestation	2 weeks post-partum (pp)	2* months pp	4 months pp	6* months pp	9 months pp	12* months pp	18* months pp	24* months pp
Self-Report Interviews											
1. Maternal Demographics	15 min	X	X	X	X		X		X		X
2. Child Feeding Assessment [70]	10–20 min			X	X	X	X	X	X	X	X
3. Maternal Beverage Intake Questionnaire [70]	5 min	X	X		X		X		X	X	X
4. U.S. Household Food Security Survey [71]	3–10 min	X			X		X		X	X	X
5. Brief Infant Sleep Questionnaire (BISQ) [72]	5 min				X		X		X	X	X
Self-Reports											
6. Current Eating Environment Assessment	5 min				X		X		X		X
7. Infant Responsive Feeding Scale [73]	5 min				X		X		X		
8. Toddler Responsive Feeding Scale [73]	5 min										X
9. Baby Eating Behavior Questionnaire [74]	5 min						X				
10. Children’s Eating Behavior Questionnaire – Toddler [75]	5 min								X		
11. Perceptions of Growth Scale [76]	3 min				X		X		X		X
12. Child Physical Activity Assessment	5 min				X	X	X	X	X	X	X
13. Knowledge Questionnaire - Maternal Nutrition, Feeding and Physical Activity Practices	10 min	X			X		X		X	X	X
14. Water Availability Assessment [77]	5 min	X			X		X		X		X
15. Perceived Stress Scale [78, 79]	3 min	X			X		X		X	X	X
16. Centers for Epidemiologic Studies Depression Scale-Revised (CESDR-10) [80]	5 min	X	X	X	X		X		X	X	X
17. Alcohol, Smoking and Substance Involvement Screening Test (ASSIST) [81, 82]	5 min	X							X		X
18. Mastery Scale [83, 84]	5 min	X			X		X		X		X
19. Infant Temperament [76]	3 min				X	X	X	X			
20. Brief Infant Toddler Social Emotional Assessment (BITSEA) [85]	10 min								X	X	X
21. Participant Satisfaction Questionnaire	10 min									X	
22. Injury Assessment	10 min	X							X	X	X
Observations Completed by Study Staff											
23. Toddler Physical Activity Assessment (Accelerometer) [48]	10 min									X	X
24. Maternal Height and Weight	5 min	X			X*				X*		X
25. Child Weight and Length	5 min			X	X	X	X	X	X	X	X
26. Home Safety Environment Scan	5–30 min		X	X				X	X		X
Specimen Collection											
27. Blood Sample Collection – Mothers	5 min			X (delivery)			X				
28. Blood Sample Collection – Infants	5 min			X (delivery)			X		X		
Process Form Completed by Study Staff											
29. Session Summary Form	No participant time burden	ADMINISTERED AT ALL VISITS									
Medical Chart Reviews											
30. Maternal Medical Chart Review	No participant time burden	THROUGHOUT THE STUDY PERIOD									
31. Child Medical Chart Review											
Total Participant Time Burden** (min):		58 min	30 min	75 min	104 min	28 min	114 min	43 min	151 min	98 min	151 min

*Maternal weight only will be collected at these time points

**The 2-month, 6-month, 12-month, 18-month and 24-month assessments are completed in two sittings, to reduce participant burden. The interviews and observations are completed in the first sitting, and the self-report assessments are completed one week later

Self-reports, interviews, and observations are conducted in participants’ homes or another private location, with data recorded in tablet computers. Medical chart reviews will be completed independently by study staff after 24 months postpartum. Blood samples are collected in the hospital at delivery and in participants’ homes or another private location at two other time points (6 and 12 months postpartum). If it is not possible to collect maternal/infant blood in the hospital at delivery, maternal blood may be collected between 36 weeks gestation and delivery, and infant blood may be collected up to one week after delivery. Blood samples are being analyzed for: fasting glucose, insulin, leptin, adiponectin, lipid panels, and C-reactive protein. All study participants will be assessed at 11 time points: baseline (< 32 weeks gestation), 36 weeks gestation, delivery (blood sample collection only), 2 weeks, 2 months, 4 months, 6 months, 9 months, 12 months, 18 months, and 24 months postpartum. Family Health Liaisons (FHLs) work together with Independent Evaluators (IEs) to administer assessments to both groups *with the exception of the baseline assessment* (conducted by FHLs only). IEs are blind to participants’ randomization status and conduct key interview and observational assessments to ensure objective data collection. IEs also help study staff complete medical chart reviews.

Participants receive a gift card or gift package upon completion of the following assessment time points: baseline, 36 weeks gestation, 2 weeks postpartum, and 2, 6, 12, 18, and 24 months postpartum. Total possible remuneration for participants in the study is $160 in gift cards and a gift package valued at $10 for participant in assessments. In addition, enrolled mothers will be given a $15 gift card for each blood sample collected from the mother or infant. This occurs at three time points for infants (delivery, 6 months, and 12 months) and two time points for the mothers (delivery and 6 months). Total possible remuneration for participants in the study is $75 in gift cards for specimen collection. Study participants can receive up to $235 in gift cards and a gift package valued at $10 if they participate in all assessments and blood collections.

### Outcomes

Selected measures are being used to assess intervention impact on the following primary and secondary outcomes.

#### Primary outcomes

1. Group differences in percentage of mothers who meet breastfeeding and complementary feeding recommendations and percentage of mothers who introduce SSBs over time as assessed by the Child Feeding Assessment. From 2 weeks to 24 months pp., mothers are asked questions from an adapted version of the Pre-School-Aged Beverage Intake Questionnaire (BEVQ-15) in addition to items developed by the study team and based on previous studies conducted by the co-investigators. The assessment will be used to assess feeding practices, duration of exclusive and non-exclusive breastfeeding, timing of introduction of complementary foods and types of first foods, and introduction of SSBs.
2. Group differences in mean scores for infant feeding style subscales assessed using the Infant Feeding Behavior Questionnaire, which asks mothers to indicate how often they engage in specific feeding behaviors to assess maternal feeding styles from 2 month to 12 months pp. There are five subscales that are used to assess maternal feeding style: Responsive, Forceful, Restrictive, Indulgent, and Uninvolved. There is no total scale score. The assessment is valid and reliable. This 24-item scale is scored with a 3-point Likert scale. A mean score is calculated for each subscale, with a range of 0 to 2. The range of the 3-point Likert scale is as follows: 0 (never), 1 (sometimes), 2 (always). Higher scores reflect higher levels of feeding style indicated by a given subscale.
3. Group differences in in mean scores for toddler feeding style subscales assessed using the Toddler Feeding Behavior Questionnaire, which asks mothers to indicate how often they engage in specific feeding behaviors to assess maternal feeding styles at 24 months pp. There are five subscales that are used to assess maternal feeding style: Responsive, Forceful, Restrictive, Indulgent, and Uninvolved. There is no total scale score. The assessment is valid and reliable. This 27-item scale is scored with a 3-point Likert scale. A mean score is calculated for each subscale, with a range of 0 to 2. The range of the 3-point Likert scale is as follows: 0 (never), 1 (sometimes), 2 (always). Higher scores reflect higher levels of feeding style indicated by a given subscale.
4. Group differences in children’s consumption of fruit and vegetable intake from SSBs, snacks, and desserts over time as assessed by the USDA Household Food Security Survey. From 6 months to 24 months pp., mothers are asked questions about her child’s fruit and vegetable consumption beginning at 6 months postpartum and at all subsequent time points. These items have been added to a standardized 18-item USDA food security survey, the U.S. Household Food Security Survey, that measures household food security. Fruit and vegetable consumption will be measured by asking mothers how much and how often her child eats fruits and vegetables and whether she feels she is able to provide her child with the fruits and vegetables he or she needs. To obtain weekly fruit and vegetable servings consumption, the number of times per week fruits or vegetables are consumed will be multiplied by the number of servings consumed each time. Serving size will be age adjusted.
5. Group differences in children’s PA levels as assessed by accelerometry. At 18- and 24-months pp., children’s PA will be measured objectively using accelerometry. We will use the Actical accelerometer, a small waterproof device (28x27x10mm) weighing 17 g, which is omnidirectional (sensing motion in all planes) and integrates the degree and intensity of motion. We will use procedures co-investigators have used successfully in previous studies of young adolescents, toddlers and their mothers, placing the accelerometer on the non-dominant ankle with a non-removable, reinforced hospital band worn next to the skin, under socks, for 7 consecutive days. Accelerometers will be attached on the day of the 18- and 24-month assessment battery and removed one week later. Data will be collected in 1-min epochs. The time-stamped data will be examined. Summary statistics will include average and total activity counts and minutes in MVPA.
6. Group differences in children’s reported PA, screen time and other sedentary activities over time as assessed by the Child Physical Activity Assessment from 2 months to 24 months pp. This 14-item self-report assessment includes questions about tummy time, crawling, walking, sedentary behavior and screen time for infants and toddlers. Questions were compiled based on a body of literature related to assessing infant/toddler physical activity.
7. Group differences in children’s mean BMI z-scores over time (2 weeks to 24 months pp) as assessed through child weight and length measurements over time. Description: Child weight (to the nearest ounce) and recumbent length (to the nearest 1/8 in.) are measured using a digital scale and a recumbent measuring board (in accordance with IHS guidelines). All measurements will be taken in triplicate, removing the most disparate measurement, and averaging the remaining two. Averages will be used to calculate BMI z-scores using age- and sex-specific WHO Child Growth Standards.

#### Secondary outcomes

1. Group differences in levels of maternal stress over time as assessed by the Perceived Stress Scale 4 (PSS-4). Completed by mothers from pregnancy to 24 months pp., the 4-item questionnaire assesses maternal stress. It is scored with a five-point Likert scale. The range of the five-point Likert scale is as follows: 0 (never), 1 (almost never), 2 (sometimes), 3 (fairly often), 4 (very often). We will reverse scores for items 2 and 3. On these questions, the scores will be as follows: 4 (never), 3 (almost never), 2 (sometimes), 1 (fairly often), 0 (very often). Scores for each item will be summed to get a total score. The lowest score is 0 and the highest score is 16. Higher scores are correlated to more stress (worse outcome).
2. Group differences in depression scores over time (pregnancy to 24 months pp) as assessed by the Centers for Epidemiological Studies Depression Scale-Revised-10 (CESDR-10). The 10-item questionnaire is a validated adapted version of the CESD-R (which has been utilized to assess depression with Navajo mothers) to screen for depression in adolescents. The questionnaire asks participants to rate how often over the past two weeks have they experienced symptoms associated with depression, such as restless sleep, poor appetite, and feeling sad. Response options range from 0 to 4 for each item, with 0 = *not at all or less than 1 day in the last 2 weeks*, 1 = *1–2 Days*, 2 = *3–4 Days*, 3 = *5–7 Days*, 4 = *Nearly every day for 2 weeks*. Scores for each item will be summed to get a total score. Scores range from 0 to 40, with high scores indicating greater depressive symptoms (worse outcome). A CESD-R score of 8 or greater indicates
3. Group differences in alcohol and drug use over time (pregnancy to 24 months pp) as assessed by the Alcohol, Smoking and Substance Involvement Screening Test (ASSIST). Adapted from the WHO ASSIST questionnaire covering 10 main substance groups, this 15-item questionnaire screens for all levels of problem or risky substance use (alcohol, illegal drugs, and prescription drugs). A risk score is provided for each substance, and scores are grouped into low, moderate, or high risk. Only items 2–7 and 9–14 are scored. Each of these questions has a set of responses to choose from, and each response has a numerical score. The scores from questions 2–7 are added together, and the scores from questions 9–14 are added together to produce an ASSIST risk score for each substance that falls into one of three categories: low, moderate, or high substance-related risk. Participants with risk scores 3 or less (10 or less for alcohol) are at lower risk of problems related to their substance use. Participants scoring between 4 and 26 (11 and 26 for alcohol) are at moderate risk of health and other problems and may be experiencing some of these problems right now. A score of 27 or higher for any substance suggests that the participant is at high risk of dependence or is dependent on that substance and is probably experiencing health, social, financial, legal, and relationship problems as a result of their substance use.
4. Group differences in how infant biologic measures of metabolic health (fasting glucose, insulin, leptin, adiponectin, lipids panels, and c-reactive protein) at delivery and 6 months postpartum correlate to maternal biologic measures of metabolic health. Blood specimens are collected from mothers at delivery and 6 months postpartum. Blood specimens are collected from infants at delivery (cord blood), 6 and 12 months postpartum. Laboratory testing will be completed to assess levels of fasting glucose, insulin, leptin, adiponectin, lipids panels, and c-reactive protein in mothers and babies to examine how infant metabolic health relates to maternal metabolic health at delivery, and whether there are between group differences in this relationship over time. In all cases, higher levels indicate poorer metabolic health.
5. Group differences in infant metabolic health at delivery, 6 and 12 months postpartum. Blood specimens are collected from infants at delivery (cord blood), 6 and 12 months postpartum. Laboratory testing will be completed to assess levels of fasting glucose, insulin, leptin, adiponectin, lipids panels, and c-reactive protein in infants to examine whether there are between group differences in infant metabolic health at delivery and over time (delivery to 12 months postpartum). In all cases, higher levels indicate poorer metabolic health. In addition, results will be used to determine whether infants are insulin resistant and/or leptin insensitive (these will be dichotomous outcomes) and between group differences will be assessed at delivery and over time (delivery to 12 months postpartum).
6. Examination of whether group differences in infant metabolic health at delivery, 6 and 12 months postpartum are moderated or mediated by sociodemographic, biological (e.g. pre-pregnancy BMI, gestational weight gain, gestational diabetes, etc.), and psychosocial characteristics of mothers at baseline). Blood specimens will be collected from infants at delivery (cord blood), 6 and 12 months postpartum. Laboratory testing will be completed to assess levels of fasting glucose, insulin, leptin, adiponectin, lipids panels, and c-reactive protein in infants. In all cases, higher levels indicate poorer metabolic health. Analyses will be conducted to determine whether between group differences in metabolic health at delivery and over time (delivery to 12 months postpartum) are moderated or mediated by sociodemographic, biological, and psychosocial characteristics of mothers at baseline.

### Quality assurance

To ensure the quality of the consent delivery, all recruitment staff have been certified to consent participants. Certification occurs after peer role playing and practice at the site, with a goal of role playing the informed consent process at least three times before being certified.

To ensure the fidelity to the intervention and quality of the curriculum delivery for both the intervention and control groups, the FHCs (who only deliver lessons to intervention group) and FHLs (who deliver lessons to control group and administer self-report assessments for both intervention and control) complete a knowledge test for each lesson and complete two role plays.

for each lesson before delivering the lesson to a participant. In addition, they are observed in person on a quarterly basis, and all lessons are audio recorded so that a random 10% of recordings can be reviewed and rated for fidelity.

### Quality control of data management

Data assessments are initially checked for quality at the field level. All data are directly entered into an electronic database using the REDCap mobile App on iPads by the FHL, IE, or the participant herself. Data are uploaded at the end of each day to the REDCap web-based database. The data management plan includes quality control at the field level and in Baltimore. Study coordinators follow data verification procedures on a weekly, bi-weekly, and quarterly basis to generate information needed to identify specific errors and missing data. Data are currently being uploaded into a secure, HIPAA-compliant server on a weekly basis by the Baltimore-based coordinator. Data are exported out of REDCap for quality control routines in Stata. All Stata data sets are stored on a JHU secure server. All errors or missing data identified are noted in a log and communicated with study coordinators. The field coordinator creates necessary queries in REDCap that field staff respond to log data corrections.

### Sample size calculation and statistical analysis

Sample size was powered to detect meaningful differences between the intervention (FSN + OSC) and control group (OSC + IPE) across six primary outcomes: 1) percent ever breastfed, 2) percent exclusively breastfed through 6 months of age, 3) mean duration of breastfeeding (at 12 months postpartum), 4) percent ever introduced sugar sweetened beverages at 12, 18 and 24 months, 5) mean score on responsive feeding scale (5-point scale – evaluated at 2, 4, 6, 9, 12, 18 and 24 months postpartum), and 6) mean BMI z-score at 24 months of age. A sample size of 338, or 169 per group, was deemed sufficient to detect meaningful differences across all outcomes, taking into account an estimated 16% loss-to-follow-up at 24 months postpartum (based on the previous FS trial) [54] and ensuring at least 80% power and significance level of 5%. Sample sizes and power were calculated using Stata 13’s power command (two means for continuous variables and two proportions for binary variables) (Table 3). The only exception among the primary outcomes was BMI z-score. Power simulation was conducted using a random draw and random treatment assignment from real site population data. The simulation varied the treatment effect and was run 10,000 times for each treatment effect level from which power was calculated. Simulation was also used to calculate power for moderator analyses. We hypothesize that maternal psychosocial factors, food/water insecurity and constrained physical activity environment will moderate the impact of the intervention on maternal feeding practices and healthy weight knowledge/behaviors. We also hypothesize that food/water insecurity and constrained physical activity could moderate the impact of improved maternal feeding practices and healthy weight behaviors on child diet and physical activity. While we will not have the power to examine interaction terms for all outcomes, we will have > 80% power to detect meaningful difference of differences in breastfeeding duration (48 days) and responsive feeding scales (0.4 to 0.6). The interaction terms for other outcomes will be explored as hypothesis generating to determine trends that would warrant further study with larger samples sizes.

**Table 3:** Sample Size Calculations

Outcome Measures	Detectable Differences
% Mothers initiated breastfeeding	14%
% Exclusive breastfeeding through 6 months of age	15%
Mean breastfeeding duration	23 days
% Introduced SSBs	13%
Infant/Toddler Responsive Feeding Scale:	
• Responsive • Forceful • Indulgent • Restrictive • Uninvolved	0.20 points 0.24 points 0.22 points 0.20 points 0.32 points
BMI z-score	0.33 (SDs)

Blood samples are collected from all mothers prior to delivery, and cord blood is collected from all infants at delivery (*N* = 338 mother/infant pairs). Blood samples are also collected from a subset of 72 mother/infant pairs at 6 months postpartum, and 260 infants at 12 months postpartum. Reduced sample sizes at 6 and 12 months postpartum are related to time and budget constraints. These sample sizes will allow us to examine whether mother/infant biologic metrics are correlated at each time point and to build predictive models for infant biologic outcomes. Additionally, *n* = 72 mother/infant pairs with baseline and 6-month follow up will allow us to detect meaningful differences in the related biometric measures by maternal characteristics and/or maternal/infant behaviors (standardized mean detectable differences = 0.68), taking into account 4% loss-to-follow-up at 6 months postpartum (based on previous Family Spirit trials) and ensuring 80% power and significance level of 5% [69]. Finally, a sample size of *n* = 260 infants at 12 months will allow us to detect meaningful between intervention group differences in infant biometric outcomes (standardized mean detectable differences = 0.38), taking into account 16% loss to follow-up at 12 months postpartum and ensuring 80% power and a significance level of 5%.

#### Primary aims analysis

We will conduct intent-to-treat analyses to estimate effects of the FSN + OSC intervention compared to the OSC + IPE control condition on early childhood obesity and related risk factors. Separately, we will examine the effects of intervention dose and conduct a “completer analyses” on subjects receiving minimum intervention dose (> 50% of lessons). Analyses will be adjusted for multiple comparisons using Bonferonni corrections.

Demographic and outcome variable distributions will be examined to assess whether comparability between intervention groups at baseline was achieved through randomization. For time discrete outcome measures, t- tests or chi-square tests will be used to test whether the intervention impacted each outcome in unadjusted analyses. Multiple linear or logistic regression will be used to test the impact of the intervention on each outcome, controlling for baseline demographic characteristics where necessary and examining potential moderators in secondary analyses. For time varying outcome measures we will examine the impact of the intervention by analyzing between group differences at each time point using t-tests and multiple regression analyses. For those outcomes with sufficient power, multi-level mixed-effects regression with a random effect at the individual level and appropriate variance structures, and an interaction term for treatment x child age will be used to examine change over the course of the intervention period for the intervention and control groups.

#### Secondary aims analysis – Biologic measures

Descriptive analyses, followed by Pearson’s pairwise correlation analyses will be conducted to examine maternal/infant correlations separately at delivery, 6 and 12 months for each biomarker (fasting glucose, insulin, leptin, adiponectin, lipids, and C-reactive protein). The ratio of maternal/infant biometrics and infant biologic metrics alone will be examined by time point and stratified to examine whether there are differences by maternal baseline characteristics (delivery, 6 and 12 months) and/or maternal/infant behaviors (6 and 12 months only). Infant multiple regression predictive models will be developed for each biologic outcome separately at delivery, 6 and 12 months to determine which maternal characteristics and/or behaviors (6 and 12 months only) are predictive of infant biomarker levels at each time point. Mixed effects models with a random effect at the family level will be used to adjust for mother/infant clusters. Mixed effects models adjusted for repeated measures, with appropriate correlation structures and a random effect at the family level will be used to examine how the maternal/infant ratio of each biomarker as well as infant biomarkers alone change between delivery, 6 and 12 months postpartum. Multiple regression predictive models will be developed for each biologic outcome to examine whether changes between delivery, 6 and 12 months differ by maternal characteristics and/or maternal/infant behaviors. Interaction terms between covariates and infant age will be included where appropriate. All models will be controlled for study group (intervention begins before delivery). Finally, infant biologic outcomes will be compared by study group at 6 and 12 months postpartum using t-tests. Multiple linear regression will be used to control for confounders/effect modifiers. Analyses will also be conducted at 12 months postpartum to determine whether the impact of the intervention on behavioral or physiologic outcomes was moderated by infant metabolic health at delivery, using multiple linear or logistic regression as appropriate. An interaction term between study group and each biologic outcome will be included in separate models.

## Discussion

This study was designed as a response to the call for childhood obesity research beginning in pregnancy and extending through the most critical developmental time period (birth-24 months of age) in children’s lives. To the authors’ knowledge, this study is the first to examine obesity prevention among NAs from pregnancy until the child’s second birthday. It is also the first to combine biologic longitudinal measures of metabolic health for mothers and infants with a home-visiting behavioral intervention.

This study has many strengths. It is using the most rigorous evaluation design (RCT) to measure intervention impact. Its assessment battery includes validated self-reports, observational and anthropomorphic measures, medical chart reviews, and biological samples to understand impacts on psychosocial, behavioral, physical and biological levels. The intervention was designed through an iterative community-based participatory approach which blends state-of-the-science early childhood diet and physical activity recommendations with valued NA cultural practices from participating communities. Employment of paraprofessional Native home visitors to teach the intervention ensures further cultural competency in intervention delivery, while offering a sustainable intervention design in communities that have a paucity of professional providers and structural and transportation barriers to clinical care and agency-based parent education. Finally, the inclusion of content to address mother’s psychosocial stressors that could impede positive feeding and parenting practices and focus on the moderating effects of water and food insecurity round out what is a comprehensive, multi-level approach necessary to addressing early childhood obesity in low resource, historically disenfranchised communities.

## Limitations

While the study uses a comprehensive mixed-methods assessment battery, it lacks observational measures of child feeding practices. The design relies on maternal self-reports via interview of child feeding practices because the communities felt this method was more culturally-appropriate than video-taping child feeding. However, the use of IEs to conduct self-report interviews has been a way for the study team to reduce bias in reporting on these measures. IEs are trained extensively in the self-report interview measures. Since IEs are blind to participant randomization status, they are not aware of what participants should and should not know based on intervention and control education. Response bias is also a concern for other maternal self- and parent-report measures. However, for more sensitive topics (i.e. drug/alcohol use, depression), the use of REDCap for maternal self- and parent-reports mitigates interviewer bias. Further, a past Family Spirit intervention found that computer-assisted data collection was successful in eliciting sensitive information from mothers in both groups (control and intervention) in the same study communities [54]. In addition, because this study is being done with Southwestern tribal communities, results may not be generalizable to other tribal and non-tribal communities. However, the participating communities differ in culture, language, population density, degree to which they are rural or semi-suburban, diet, and beliefs—offering notable heterogeneity to the study population. Further, a past Family Spirit intervention designed with the same Southwestern communities has been shown to be culturally-adaptable and acceptable to diverse tribal and non-tribal communities across the United States.

If proven effective, Family Spirit Nurture will yield new innovations for tribal communities and the home-visiting field more broadly to overcome the nation and world’s public health crisis related to early childhood obesity. While Congress reauthorized the Maternal Infant Early Childhood Home-Visiting (MIECHV) Program legislation in February of 2018 to scale what are now 20 federally endorsed home-visiting models to states and tribes, none of the current home-visiting models have demonstrated impacts on preventing early childhood obesity among NAs. Because the original Family Spirit program, one of the 20 federally endorsed models, is already operating in over 120 urban, rural, and tribal communities across 19 states, with further expansion monthly, the Johns Hopkins Center for American Indian Health study team has a ready network and defined channels for scaling FSN if significant effects are demonstrated.

## Abbreviations

AAP: American Academy of Pediatrics
BMI: body mass index
CVD: cardiovascular disease
DSMB: Data Safety and Monitoring Board
FHC: Family Health Coach
FHL: Family Health Liaison
FS: Family Spirit
FSN: Family Spirit Nurture
IE: Independent Evaluator
IHS: Indian Health Service
IPE: Injury Prevention Education
JHCAIH: Johns Hopkins Center for American Indian Health
MIECHV: Maternal, Infant, and Early Childhood Home-Visiting
NA: Native American
OPRE: Office of Planning, Research, and Evaluation
OSC: Optimized Standard of Care
PA: physical activity
RCT: randomized controlled trial
REDCap: Research Electronic Data Capture
SES: socioeconomic status
SSB: sugar-sweetened beverage
US: United States
WHO: World Health Organization
WMAT: White Mountain Apache Tribe
